# Development of the Nephrotic Syndrome Symptom and Impact Patient Reported Outcome (NephroSSI-PRO) measure for use in adults and adolescents with primary glomerulopathies

**DOI:** 10.1186/s41687-025-00937-7

**Published:** 2025-08-21

**Authors:** Joshua Maher, An-Qi Hu, Sagar U. Nigwekar, Julie Lin

**Affiliations:** 1 Sanofi, Reading, UK; 2 Sanofi, Cambridge, MA USA; 3 Division of Nephrology, Department of Medicine, Massachusetts General Brigham, Boston, MA USA

**Keywords:** Clinical outcome assessments, Disease impacts, Disease symptoms, Health-related quality of life, Nephrotic syndrome, Patient-reported outcomes

## Abstract

**Background:**

Patient reported outcome (PRO) instruments are important for assessing the effects of new therapeutics; however, no PRO measures have been specifically developed for use in Nephrotic Syndrome (NS) with documented evidence of content validity.

**Methodology:**

The Nephrotic Syndrome Symptom and Impact Patient Reported Outcome (NephroSSI-PRO) tool was developed following a literature review, concept elicitation (CE) interviews with patients with NS and clinicians, item generation, and cognitive debriefing (CD) interviews with patients with NS. The CE interviews were conducted with four nephrologists and among adults (aged ≥ 18 years) and adolescents (aged 12–17 years) diagnosed with NS.

**Results:**

In total, 45 adult and adolescent patients were interviewed across all CE and CD interviews. In the adult CE interviews, the most frequently reported symptoms were swelling in the feet/legs (*n* = 14/16), general tiredness (*n* = 13/16), change in appetite (*n* = 13/16), foamy urine (*n* = 12/16), and shortness of breath (*n* = 12/16); impacts included emotional health, physical function limitations, sleep difficulties, and social/lifestyle limitations (*n* = 13/16). In adolescents, the most frequently reported symptoms were tiredness/fatigue (*n* = 13/15), stomachache (*n* = 11/15), headache (*n* = 8/15), foamy/frothy urine (*n* = 7/15), swelling in face/eyes (*n* = 6/15), swelling in legs/ankles (*n* = 5/15), and change in appetite (*n* = 5/15), while the most common impacts were impact on schooling (*n* = 10/15), reduced ability to practice sports (*n* = 7/15), and impact on friendship (*n* = 7/15). A conceptual model was developed and used to inform item generation for the NephroSSI-PRO. During CD interviews, there were no challenges in understanding and relating to instrument instructions, items, and response options.

**Conclusions:**

We have developed a PRO instrument to evaluate the symptoms and impacts of NS in adult and adolescent patients. In this study, the evidence from a literature review, and direct patient input from CE and CD support its content validity. Further work supporting the instrument’s validity will be performed in a future clinical trial.

**Supplementary Information:**

The online version contains supplementary material available at 10.1186/s41687-025-00937-7.

## Introduction

Evaluating the disease burden of nephrotic syndrome (NS) can be achieved using a patient-reported outcomes (PRO) instrument. NS has a significant impact on various aspects of health-related quality of life (HRQoL), highlighting the importance of using PRO instruments to evaluate disease burden. To be considered fit-for-purpose in a specific context of use, a PRO must meet established regulatory and evidentiary standards [1]. Existing PRO instruments commonly used in kidney diseases [2–9] were not developed specifically for patients with NS; either lack psychometric validity or fail to capture the range and granularity of NS-specific concepts required to comprehensively measure the patient experience of NS [10, 11]. For example, the Patient-Reported Outcomes Measurement Information Systems (PROMIS) measures in mobility, pain interference, fatigue and mental health lack significant anchoring to clinically-defined NS disease activity and remission status, and the authors highlighted that there is an opportunity to develop a disease-specific HRQoL measure for patients with NS [12]. 

Further, many existing instruments used in NS populations exhibit considerable variability in recall periods, ranging from 24-hours up to four weeks, but often without clear disease-specific rationale. Given that symptoms of NS can often evolve rapidly over time and are often characterized by fluctuation and periods of relapse and remission [1, 13–15], it is crucial that recall periods align with the clinical course of the disease to support accurate and meaningful symptom reporting. Together, these limitations underscore a significant gap in adequately measuring the severity and burden of NS symptoms and impacts highlighting the importance of developing a PRO measure specifically for this patient population. This study outlines the qualitative development of a novel PRO instrument, the Nephrotic Syndrome Symptom and Impact Patient Reported Outcome (NephroSSI-PRO), for use in adult and adolescent patients with NS caused by primary glomerulopathies.

## Methods

This cross-sectional, non-interventional study involved multiple steps to identify key NS disease concepts and support PRO development via: (1) a review of literature that discussed the signs, symptoms, and impacts of NS caused by Focal Segmental Glomerulosclerosis (FSGS); (2) one-to-one qualitative concept elicitation (CE) interviews with clinicians; and (3) one-to-one qualitative CE interviews with adult patients. This was followed by instrument development and preliminary qualitative evaluation via: (4) item generation based on steps 1–3; (5) cognitive debriefing (CD) interviews with adult patients to evaluate the draft items of the novel PRO instrument; and finally, (6) hybrid CE and CD interviews with adolescent patients to evaluate the content validity of the novel PRO instrument in adolescents. Interaction with the US FDA (United States Food and Drug Administration) was sought at multiple stages throughout the instrument development process, and feedback was incorporated to inform the overall approach and ensure regulatory alignment. 

All research was conducted in accordance with the ethical principles originating from the Declaration of Helsinki, Good Clinical Practice, and regulatory requirements [16]. Study protocols and patient-facing materials were approved by Institutional Review Boards (IRBs; Quorum for the adult studies; Copernicus for the adolescent study). Informed consent was obtained from all patients and parents/caregivers of adolescent patients and participants were remunerated according to Fair Market Value rates. The sample size for adult and adolescent interviews was defined based on expected recruitment feasibility in a rare disease population, while also aiming to achieve conceptual saturation [17]. 

### Review of key literature and clinician CE interviews

A review of existing empirical literature and CE interviews with four clinicians who had clinical and research expertise in nephrology with a focus in glomerular diseases were conducted to identify disease-specific concepts (i.e. signs, symptoms, and impacts). For more information, see Supplementary Tables 1 and 2.

### Adult patient CE interviews

Health Research Associates, Inc. (HRA), a Clinical Research Organization (CRO) partnered with three outpatient nephrology clinical sites responsible for recruiting, screening and scheduling interviews for eligible adult patients (≥ 18 years) in the United States (US) with NS in 2013. A purposive sampling method was used, and individuals were deemed eligible to participate if they had a prior confirmed diagnosis via renal biopsy of FSGS, Minimal change disease (MCD), Membranous nephropathy (MN), or Immunoglobulin A nephropathy (IgAN).

Interviews lasted approximately 60 minutes and were conducted in-person by trained HRA moderators at these sites using a semi-structured interview guide to explore patients’ perspectives on the signs, symptoms, and impacts of NS that were relevant and important to patients. Interviews were audio-recorded, transcribed verbatim for coding and content analysis and analyzed using thematic analysis methods, facilitated by Atlas.ti software. Adult patients were asked to provide severity and bothersome ratings on a 0–10 numeric rating scale (NRS; 0=“none” to 10=“extremely severe” and 0=“not bothersome at all” to 10=“extremely bothersome”) for each symptom they experienced. Additionally, patients were asked to rate how difficult each impact was to cope with on a 0–10 NRS (0=“not difficult at all” to 10=“extremely difficult”).

To evaluate saturation, the 16 transcripts were organized chronologically based on the order in which the interviews were conducted, into four groups/waves of four transcripts each. Saturation of concepts was achieved when no new disease-related concepts were identified in a subsequent wave of transcripts. Concepts reported by adult patients, together with findings from the literature review and clinical expert interviews, were used to generate a conceptual model of NS.

### Item generation and instrument development

Item generation involved a review and discussion of all conceptual findings with a multidisciplinary team, including instrument development experts, nephrologists, and the study Sponsor (Sanofi). Draft items were developed based on the conceptual model and direct patient quotes extracted from the CE interviews. These items were then further scrutinized and refined with clinician input.

### Adult patient CD interviews

CD interviews were conducted in-person, lasted for approximately 60 min and audio recordings were transcribed verbatim for analysis. CD was conducted in waves of four to six patients to assess item relevance, clarity, and comprehensiveness, and the appropriateness of response options. Following each wave, items were reviewed and revised as needed and re-tested iteratively until patients comprehended the items as intended.

### Adolescent patient hybrid CE and CD interviews

A total of 15 adolescent patients (aged 12–17 years) in the US were recruited and interviewed by IQVIA, a CRO‚ in collaboration with NephCure (a patient advocacy group for rare kidney disease patients) and Global Perspectives (a patient recruitment company). Adolescent patients were recruited only if they had experienced symptoms of NS within the past six months to help mitigate recall bias in the younger population. Confirmation of diagnosis was obtained via documentation from a treating physician or submitted medical records.

Adolescent interviews followed a hybrid format, comprising both CE and CD in the same 60-minute interview, and were conducted via teleconference by trained moderators with screensharing capabilities. Interviews were audio-recorded and transcribed verbatim for analysis.

Similar to the adult CE interviews, adolescent interviews employed a semi-structured interview guide to explore the relevant and important signs, symptoms, and impacts of NS. However, in contrast to the adult CE interviews, the CE approach was streamlined for adolescent interviews due to time constraints associated with the hybrid CE/CD format and the need to prioritize CD of the NephroSSI-PRO. Instead, the focus was on identifying the most relevant and bothersome concepts. To facilitate this, participants were asked to identify only the most bothersome symptom and impact: from those they had just reported which were then ranked by frequency. Transcripts were analyzed using MAXQDA, qualitative analysis software. A similar thematic analysis approach was used to analyze the CE portion of the interviews as with the adult interviews. Although conceptual saturation was assessed, the limited time available for CE meant that achieving saturation was unlikely. To evaluate saturation, the 15 transcripts were organized into three groups of five transcripts. Concepts reported by adolescent patients informed the development of an age-specific conceptual model of NS.

For the CD component, adolescent patients were also asked about the relevance, clarity, and comprehensiveness of items, the appropriateness of response options, and the 24-hour recall period of the NephroSSI-PRO. Adolescent patients were also invited to offer more detailed feedback on an item related to “being limited in the ability to work” due to a priori concerns about the item’s relevance to adolescent patients.

## Results

### Review of key literature and clinician CE interviews

The literature review and CE interviews with clinicians led to the development of a preliminary conceptual model, which was reported by the clinicians to be generalizable to all patients with NS with primary glomerulopathy. These signs and symptoms were used to structure and develop the discussion guide for patient CE interviews. For more information, see Supplementary Tables 1 and 2 [18–27]. 

### Adult patient CE interviews

A total of 16 adult patients with NS were interviewed; the mean age was 51.1 years (standard deviation [SD] = 12.7) and just over half were female (*n* = 9/16). A majority were married or cohabiting (*n* = 12/16) and of White ethnicity (*n* = 12/16). Of the 16 patients, seven had a diagnosis of FSGS, eight had a diagnosis of MN, and one had a diagnosis of MCD. Although the inclusion criteria allowed for the inclusion of patients with IgAN as per recommendations from clinician interviews, no patients with IgAN were recruited. Six patients were resistant to steroids (three patients with FSGS and three with MN). The mean (SD) duration since the initial diagnosis of glomerulopathy was 11.6 (8.9) years (Table 1).

**Table 1:** Demographics and clinical characteristics of patients included in concept elicitation and cognitive debriefing interviews

Characteristics	Adult patients		Adolescent patients
	Concept elicitation patients (*n* = 16)	Cognitive debriefing interview patients (*n* = 14)	Concept elicitation and cognitive debriefing interview patients (*n* = 15)
Age, years, mean (SD)	51.1 (12.7)	50.9 (13.2)	15.7 (1.4)
Female, n (%)	9	7	7
***Marital status***,*** n (%)***			
Married or cohabiting	12	8	NA
Divorced	3	3	NA
Separated	1	1	NA
Widowed	-	1	NA
Never married	-	1	NA
***Race***,*** n (%)***			
White	12	11	6
Black or African American	4	3	2
Two or more races	-	-	1^a^
Prefer not to say	-	-	6
***Current Employment Status***,*** n (%)***			
Employed for wages	8	7	NA
Self-employed	2	2	NA
Out of work for more than 1 year	1	1	NA
Retired	2	1	NA
Unable to work	3	3	NA
***Highest level of education***,*** n (%)***			
Currently attending middle school (Grades 6–8)	-	-	2
Currently attending high school (Grades 9–12)	-	-	13
Less than High School	1	2	
High School	7	7	
College	6	4	
Graduate or Professional School	2	1	
***Nephrotic Syndrome Subtype***,*** (%)***			
Steroid resistant FSGS	3	2	1
Steroid resistant MN	3	2	-
Non-steroid resistant FSGS	4	3	8
Non-steroid resistant MN	5	6	-
Non-steroid resistant MCD	1	1	4
Years since initial diagnosis of glomerulopathy, mean (SD)	11.6 (8.9)	11.9 (10.3)	9.8 (7.0)^b^
Years since renal biopsy confirmation of glomerulopathy, mean (SD)	5.9 (5.2)	5.2 (5.7)	-

SD: Standard Deviation; FSGS: Focal segmental glomerulosclerosis; MN: Membranous nephropathy; MCD: Minimal change disease

In the CE interviews, the most frequent symptoms experienced and reported by adult patients were swelling in the feet/legs (*n* = 14/16), general tiredness (*n* = 13/16), change in appetite (*n* = 13/16), foamy urine (*n* = 12/16), and shortness of breath (*n* = 12/16) (Table 2). Quotations from patients describing these symptoms are included in Table 3.

**Table 2:** Summary of adult concept elicitation results: symptoms

Adult Symptom Domains and Concepts	Adults (*n* = 16)		
	Number of patients mentioning symptom	Mean severity rating	Mean bothersome rating
***Urinary symptoms***	***14***		
Foamy urine	12	7.9	4.6
Frequent urination	6	-	10
Black/blood in urine	1	-	-
***Digestive symptoms***	***14***		
Appetite	13	3.7	7.3
Nausea	5	5.8	5.5
Constipation	1	10.0	10.0
***Edema symptoms***	***14***		
Swelling in the feet/legs	14	7.1	6.5
Swelling in general	7	9.3	4.8
Swelling in the face	6	-	-
Water weight	6	-	-
Tightness	6	-	-
Swelling in hands/arms	3	-	-
Bloating	2	4	5
Stretch marks	2	-	-
***Energy difficulties***	***13***		
General tiredness	13	8.0	8.3
Physical tiredness	7	-	-
Low/no energy/weakness	5	-	6.5
***Pain symptoms***	***14***		
Headache	7	7.6	4.3
Localized pain in the back	6	8.8	8.2
Localized pain in the Feet/Legs	6	7.5	7.5
General body pain	5	10	5.5
Cramping	4	9.3	6.6
Other localized pain	4	-	8.3
Localized pain in chest	3	-	-
Other localized pain (back of head/neck, esophagus, groin, sides)	4	-	8
***Respiratory symptoms***	***13***		
Shortness of breath	12	7.3	6.8
Difficulty breathing	5	7.4	5.3
Other (cough, difficulty swallowing, hoarseness, pneumonia)	5	7.5	7.0
Wheezing	3	-	-

**Table 3:** Selection of adult patient descriptions of disease symptoms

Symptoms	Description
***Urinary symptoms***	
Foamy urine	“A little bit of foamy urine” “Every time I pee, it’s really foamy”
Frequent urination	“I go three or four times during the day. It is an inconvenience” “It hurts for me to urinate constantly”
***Digestive symptoms***	
Appetite	“I can’t eat no more than what a 3-year-old would eat at one time” “I don’t eat usually a lot in the day”
Nausea	“I feel nauseous, like I have air in my stomach” “I’ll just feel sick to my stomach, like I have to throw up”
***Edema symptoms***	
Swelling in the feet/legs	“I could start to feel it on the side of my work shoe, they’re slightly more swollen than normal” “I swell mainly in my ankles and feet”
Swelling in general	“I swell a lot, fluid build-up in my chest, my stomach” “I was swelling, I had so much water I looked kind of puffy”
Swelling in the face	“I wake up with my eyelids all puffy” “My face started to swell on and off”
Water weight	“I had gained 40 pounds of water, I looked like a marshmallow” “I swell, lot of fluid buildup, I’m 15 pounds heavier than I usually am”
Tightness	“I can flex my knees and feel the tightness” “It affects my ability to walk a little bit because my ankles are so tight”
***Energy difficulties***	
General tiredness	“I am always tired” “I get very fatigued, very sleepy”
Physical tiredness	“I feel like I’ve run a marathon” “My body feels tired like I just want to lie down and relax”
Low/no energy/weakness	“I feel like a battery running down” “I just don’t have the energy that I used to have”
***Pain symptoms***	
Headache	“I experienced headaches really bad” “I had a bad headache, I woke up with it”
Localized pain in the back	“I get up some days with my back hurting” “Just had really bad pains like in the back for like a day or two”
Localized pain in the Feet/Legs	“I’ll get cramps in the calf of my legs” “My legs and lower legs cramp, I get Charley horses”
General body pain	“I have body aches and pains” “All over, could be hurting bad”
Cramping	“I get cramping from time to time” “It’s just like a moderate body cramp of a 2 or 3 maybe”
Other localized pain	“I had a lot of pain and soreness in my sides” “I will uncontrollably hiccup and that hurts, my esophagus feels raw”
Other	“I feel like I have to move my legs, they feel like icky” “I’ll be so stiff in the morning time”
***Respiratory symptoms***	
Shortness of breath	“I am breathing in as deep as I can, but I’m not getting enough air” “I can’t get enough air in”
Difficulty breathing	“I don’t know how to relate it to what caused the breathing to be so labored” “I couldn’t breathe”
Other	“I can’t swallow” “I have a coughing spell”

Among these symptoms (on the NRS of 0–10, higher scores = more severe/bothersome), cramping (mean = 9.3), general body pain (mean = 10.0), and generalized swelling (mean = 9.3) received the highest severity ratings, and frequent urination (mean = 10.0), general tiredness (mean = 8.3), weight gain (mean = 8.3), and back pain (mean = 8.2) received the highest bothersome ratings (Table 2).

The most frequent impacts reported by adult patients included: emotional health, physical function limitations, sleep difficulties, and social/lifestyle limitations, with > 13 adult patients reporting all four of these impact domains. The impacts rated as the most difficult to cope with (on the NRS of 0–10, higher scores = more difficult) were mood swings (mean = 9.3), fear of dialysis (mean = 7.3), general physical function (mean = 8.3), walking (mean = 8.8), work impacts (mean = 8.6), and fear/worry about future (mean = 8.5) (Table 4).

**Table 4:** Summary of adult concept elicitation results: impacts

Nephrotic Syndrome Impact Domains and Concepts	Adults (*n* = 16)	
	Number of patients mentioning impact	Mean difficulty rating
***Emotional health***	**16**	
Worry/fear general	13	8.5
Worry/fear of dialysis	10	7.3
Frustration	9	-
Irritability	9	3.0
Sadness	9	7.8
Poor body/self-image	7	-
Anger	7	7.0
Worry/fear financial	1	8
***Physical function limitations and restrictions***	**14**	
Walking	10	8.8
General	8	-
Stairs	5	-
Slowness	3	6
Walking other (bending, climbing, exercise, lifting, reaching)	2	-
***Social/lifestyle limitations and restrictions***	**13**	
Leisure	7	7.3
Relationships	7	6.3
Social activity	7	7.8
Clothing choice	3	-
Sexual activity	3	10
***Sleep difficulties***	**13**	
Waking to urinate	8	-
Difficulty falling asleep	7	-
Difficulty staying asleep (not due to need to urinate)	7	-
Sleep quality	5	-
Sleep position	4	-
***Difficulty in doing daily activity***	**12**	
Home	10	6.3
Work	7	8.6
General	7	-
Personal care	5	-
***Coping behaviors***	**11**	
Outlook	9	-
Dietary restrictions	8	-
Medication	7	-
Vigilant	4	-
Water consumption	4	-
Social support	3	8
Germaphobia	2	10
Sleep	2	-
Other (exercise)	1	-

Saturation was achieved for both symptoms and impacts in adult patients with no new concepts emerging in the final two waves of interviews (Supplementary Table 3). Together, data from Tables 2 and 4 comprise the refined conceptual model of NS in the adult population.

### Item generation and instrument development

Twenty-two initial items were developed through triangulation of evidence from the literature, clinical expert input, and patient interviews. These activities informed the development of a conceptual mode that guided concept selection and item generation. Items were developed to focus primarily on core symptoms and proximal impacts that are most directly linked to the disease experience. Broader, distal HRQoL impacts were limited to minimize conceptual overlap with existing instruments, reduce respondent burden, and maintain disease specificity. All items utilized an 11-point NRS, ranging from 0 to 10, and respondents were asked to indicate the severity of their symptoms and impacts at their worst in the 24 h prior to assessment.

### Adult patient CD interviews

Three waves of CD interviews were conducted; the first two waves with four patients each and the third wave with six patients. No significant issues understanding and following the instructions, items, and response options of the NephroSSI-PRO arose during Wave 1. Based on feedback from one patient in Wave 1 who found the “whole body swelling” item confusing when it preceded location-specific swelling items, the development team revised the item order to present the “whole body swelling” item after location-specific items. This change aimed to minimize cognitive burden, confusion and potential order effects by moving from specific to more general concepts. Observations during Wave 2 indicated that the revised order improved clarity for all participants. A similar change was made to the “whole body pain” item which was re-ordered to follow the location-specific pain items, further improving the logical flow and interpretability of the questionnaire.

Further revisions were made prior to Wave 3 based on feedback received from the FDA: the foamy urine item was moved to the end of the instrument to distinguish it from symptom and impact items; a new item was added to assess shortness of breath while walking; kidney disease attribution wording (“due to your kidney disease”) was removed for each item; and the Physical Function Limitations domain was expanded with three new items assessing limitations in climbing stairs, bathing or dressing, and bending over or bending down. No new issues arose during Wave 3 interviews for the original items or the new items. These revisions, along with the Wave 3 findings, resulted in the 26-item draft version of the instrument.

### Adolescent patient hybrid CE and CD interviews

In total, 15 adolescent patients with NS were included. The mean age of patients was 15.7 years (SD = 1.4); seven were female, six were male, and two of them did not disclose their gender. Six patients were White, two were Black or African American, one patient reported having two or more races, and the remaining six patients did not disclose their race. Eight participants reported their ethnicity as non-Hispanic/Latino(a) (*n* = 8/15). The majority were currently attending high school (grades 9–12) at the time of completing the interview (*n* = 13). Ten adolescent patients had a diagnosis of FSGS and five of MCD. The mean (SD) duration since the initial diagnosis of glomerulopathy was 9.8 (7.0) months (Table 1).

A total of 42 unique symptoms were reported during the interviews with adolescent patients. The most frequently reported symptoms were tiredness/fatigue (*n* = 13), stomachache (*n* = 11), headache (*n* = 8), foamy/frothy urine (*n* = 7), swelling in face and eyes (*n* = 6), swelling in legs and ankles (*n* = 5), and change in appetite (*n* = 5). Of these, tiredness/fatigue (*n* = 4), headache (*n* = 3), stomachache (*n* = 3), and swelling on whole body or in groin area (*n* = 2) were most frequently reported as the most bothersome symptoms (Table 5). Patient descriptions of the most frequently reported symptoms are available in Table 6.

**Table 5:** Summary of adolescent concept elicitation results: symptoms

Adolescent Symptom Domains and Concepts	Number of participants mentioning symptom (*N* = 15)	Reported as most bothersome symptom (*N* = 14)
***Constitutional Symptoms***		
Tiredness/Fatigue	13	4
Appetite changes	5	-
Dizziness	2	1
Physical weakness	2	-
Excessive sweating	1	-
Increased thirst	1	-
Infection	1	-
Overheated easily	1	-
Soreness/Tenderness	1	-
***Pain – by location***		
Stomachache (pain in abdomen)	11	3
Headache	8	3
Pain in legs	3	-
Back pain	2	-
Pain in pelvis area	2	1
Pain – unspecified location	2	-
Chest pain	1	-
Eye pain	1	-
Muscle pain	1	-
Pain-related swelling	1	-
***Urinary symptoms***		
Foamy/frothy urine	7	1
Spilling protein (proteinuria)	4	-
Issues with urinating – unspecified*	2	1
Pressure when urinating	1	-
***Swelling – by location***		
Swelling in face and eyes	6	-
Swelling in legs, ankles	5	-
Swelling in abdomen	2	-
Swelling in arms	2	-
Swelling – whole body	2	2
***Metabolic and gastrointestinal symptoms***		
Bloating (abdomen)	4	1
Nausea	3	1
(Abnormal) weight gain	3	1
(Abnormal) weight loss	1	-
Constipation	1	-
Diarrhea	1	-
***Pulmonary symptoms***		
Shortness of breath	2	1
Fluid in lungs	1	-
***Hematological symptoms***		
Anemia	1	-
Easily bruised	1	-
Low iron levels	1	-
***Skin-related symptoms***		
Skin tightness	1	-
Stretchmarks	1	-
***Other***		
Eating issues – uncertain type	1	-

**Table 6:** Selection of adolescent patient descriptions of disease symptoms

Symptoms	Description
*Tiredness/fatigue*	“That’s fatigue, like today I’m a little bit tired” “Some days I’m just really tired and my mom tells me I have to make sure I take care of myself more”
*Foamy/frothy urine*	“Pee’s a little bubbly” “Where my pee is very weird and strange. Maybe that is my pain in the belly has something to do with my pee, I think”
*Stomachache*	“It’s worse. Oh, and I get some stomach pain. Yeah, sometimes it’s really bad where I just feel like my head really hurts too much and that’s when I don’t go to school” “Usually like bubbly in the bottom of my stomach and it feels like it’s sitting on… Like sharp pains and it feels like it’s sitting on the bottom of my stomach, like is that the pelvis, I believe”
*Headache*	“I get headaches, and you don’t want to do stuff. You don’t want to hang out with friends when you have headaches. You don’t like doing anything with headaches, and you can’t do homework and stuff like that, so you don’t want to do anything” “If the headache is really bad, that’s the worst one. If it’s not too bad, then it’s me feeling very tired… When I have a really bad one, it pushes, I don’t know how to say this, it pushes down where I just feel like the head makes the rest of the head feel bad”
*Swelling in the face and eyes*	“Yeah, it’s a little harder to recognize me because I look really puffy in the face” “When there was a lot of fluid in my face […] [M]y cheeks were really full. My throat was swollen. It was to the point where my uvula was swollen, and I was choking on that, and that’s where the struggling to breathe came from”
*Swelling in the legs and ankles*	“My feet really hurt, and my legs were the equivalent of balloons, and I had gained a shocking amount of weight within a month. I’d gone from roughly 177 to 205…The reason why I describe it as a balloon, is it felt as if someone were to cut into me, it felt like all that fluid would come out. That’s how tight on my skin it felt” “Yeah, when I… Maybe I don’t notice it as much now because I’ve…I’m a little accustomed to it. But when I was diagnosed, before I was diagnosed a year ago, I was noticing I was swelling in my arms and my legs. That’s what kind of inclined me and my mom to actually get checked…get me checked”
Changes in appetite	“Yeah. Yeah, I mean, I was…I had some loss of appetite, but my clothes wasn’t feeling like before. My shoes were tight” “Like might not feel like eating because I have a stomachache or feel tired and have a headache”

In addition, 50 unique impacts were reported. Impact on schooling/school performance (*n* = 10), reduced ability to practice sports (*n* = 7), impact on friendship (*n* = 7), reduced attendance at social events (*n* = 6), and avoidance of social situations (*n* = 4), and dietary changes/restrictions (*n* = 5) were among the most frequently reported impacts by adolescent patients. The following impacts were reported as the most bothersome: ability to practice sports (*n* = 3), reduced attendance at social events (*n* = 2), having an overall impact on their friendships (*n* = 2), and dietary changes/restrictions (*n* = 2) (Table 7).

**Table 7:** Summary of adolescent concept elicitation patient results: impacts

Adolescent Impact Domains and Concepts	Adolescents (*N* = 15)	
	Number of participants mentioning symptom	Number of times reported as most bothersome symptom
***Role impact***		
Impact on schooling/school performance	10	-
Absenteeism	3	1
***Social impact***		
Impact on friendships	7	2
Reducing attendance at social events	6	2
Avoidance of social situations	4	1
Impact on social functioning	2	1
Making others worry	1	-
Social isolation	1	-
***Physical impact***		
Reduced ability to practice sports	7	3
Difficulty walking short distance	2	1
Inability to practice sports	3	-
Impact on vision	1	-
Need to rest more	1	-
Difficulty walking long distance	1	-
Impact on driving	1	-
Difficulty running	1	-
***Impact on diet***		
Dietary changes/restrictions	5	2
***Impact on activities of daily living (ADL)***		
Difficulty feeding oneself	1	-
Difficulty washing oneself	1	1
Difficulty dressing	1	-
General - not specified	1	-
***Impact on cognitive functioning***		
Impact on focus/concentration	3	-
Impact on memory	2	1
***Emotional impact***		
Anxiety	2	-
Apathy	2	-
Worry about disease/relapse	2	1
Annoyance	1	-
Depression	1	-
Embarrassment	1	-
Fear of death	1	-
Feeling happy	1	-
Frustration	1	1
Mood swings	1	-
***Psychological impact***		
Altered body image	2	-
Low self-esteem	1	-
Impact on sexual life	1	-
***Impact on general activities***		
Doing things slower	1	-
Pushing self to do activities	1	-
Impact on choices - cannot use transportation	1	-
Impact on choices due to accessibility for needs	1	-
Impact on hobbies	1	-
Inability to play	1	-
***Impact due to compromised immunity***		
Relapse because of sickness	1	1
Need to wear a mask in public	1	1
Hygiene restrictions	1	-
***Treatment burden***		
Keeping track of taking medication	1	1
***Sleep interference***		
Falling asleep unexpectedly	1	-
Falling asleep early	1	-
Unable to fall asleep	1	-
Poor sleep quality	1	-
***Other***		
Clothing restrictions	2	-

Saturation was not achieved for the adolescent patient interviews as new concepts were reported in the final wave of interviews (Supplementary Table 4). Together, data from Tables 5 and 7 comprise the conceptual model of NS in the adolescent population.

The 26-item NephroSSI-PRO was then cognitively debriefed item-by-item with all 15 adolescent patients. Following the in-depth item-level debriefing, patients were asked more generally about the comprehensiveness of the instrument (i.e., whether they felt there were any missing concepts). Eight participants confirmed that the NephroSSI-PRO contained all relevant concepts to their disease while three patients indicated that one or more concepts were missing from the NephroSSI-PRO (two patients thought that one concept was missing, while one patient felt that two concepts were missing). The missing concepts included: general psychological impact, impact on social life, restriction of fluid and salt intake, and swelling in groin area. The remaining four patients were not asked the broader comprehensiveness question. In three cases, the question was omitted due to time constraints, and in one case, the participant was unable to respond due to fatigue. One FSGS patient suggested a shorter, 3-question approach regarding the general impact of the disease, the extent of one’s tiredness/fatigue, and the impact of NS on general wellbeing. No other suggestions for removing concepts or items from the instrument were provided by adolescent patients.

Adolescent patients’ responses for item 25 (limited ability to work) suggested that while most were able to understand and respond to this item, the current content of this item may not be relevant for adolescent patients with NS. Based on participant feedback, item 25 was revised to include situations where adolescents may still be in school to make this item more relevant to this age demographic. Thus, the words “including schoolwork” were added to item 25 in parentheses, thereby retaining the original concept for ability to work but expanding the concept for the sake of relevance for adolescent patients. Generally, patients agreed that all questions in the NephroSSI-PRO were relevant to patients with NS.Thirteen patients were also asked about whether they would be able or willing to complete the NephroSSI-PRO daily for a week; five patients responded ‘unequivocally yes’ and five patients responded ‘yes with qualifiers’ to this question, noting some caveats regarding having to complete the NephroSSI-PRO every day, including the length of the questionnaire (n = 2), a preference for weekly completion instead of daily completion (n = 2), and the need for daily reminders (n = 1). The three remaining participants stated that they would not be able or willing to complete the questionnaire daily because it was too long (n = 2) or that they would forget (n = 1).

Following all interviews, a final version of the NephroSSI-PRO was developed for use in both adults and adolescents. The final instrument comprises 26 items covering the most important signs, symptoms, and impacts of NS reported across both age groups. Cognitive debriefing interviews confirmed that the concepts were well understood and considered relevant by both adult and adolescent populations. The response options for all items include a 0–10 numeric rating scale, with 0 being the absence of the sign/symptom/impact, and 10 being the worst manifestation of the sign/symptom/impact. All items also utilize a 24-hour recall period, and total estimated time for completion is approximately 5–10 min, allowing for approximately 16 s per item [28]. The key domains of the NephroSSI-PRO include swelling, pain, bloating, headache, fatigue, dyspnea, hunger, thirst, worry, physical limitations, limitations in activities of daily living, limitations in ability to work (including schoolwork), and foaminess of urine (see Table 8 for full list of concepts covered).

**Table 8:** Final concepts and domains covered by the Nephrotic Syndrome Symptom and Impact Patient Reported Outcome (NephroSSI-PRO) instrument

Concepts
Swelling, including in face, legs/ankles/feet, or whole body
Pain in face, back, chest, belly, legs/ankles/feet, or whole body
Bloating (in belly)
Headache
Tiredness
Physical weakness
Shortness of breath (including while walking)
Hunger
Thirst
Worry
Physical limitations, including walking, climbing stairs, bathing/dressing oneself, bending the body, and general physical activities
Limited in activities around the home
Limited in ability to work / go to school
Urine foaminess

## Discussion

In total, 45 adult and adolescent patients were interviewed across all interviews for this study. CE data confirm that adult and adolescent patients with NS experience a range of burdensome symptoms, including swelling, pain, fatigue, which in turn profoundly impacts patients’ physical functioning, emotional health, and activities of daily living [29–31]. These findings are consistent with existing qualitative literature, which has similarly identified swelling, fatigue, and pain as the prominent symptoms, along with additional symptoms such as shortness of breath, appetite changes, and foamy urine [29, 32–35]. Physical functioning is also consistently reported as the impact domain most affected across these studies, reinforcing the importance of functional limitations experienced by patients with NS.

As per regulatory guidance on the use, development, and validation of PRO instruments [1], assessment of HRQoL yields important information about the patient experience. However, validated PRO tools to assess symptoms and impacts specific to patients with NS are lacking [2–9], yet they are needed to help make informed, patient-centered treatment decisions, and to assess how a treatment changes how a patient feels and functions in clinical trials. To help bridge this gap, we have developed the NephroSSI-PRO instrument. This study represents the first step towards establishing the NephroSSI-PRO’s content validity, as defined by regulatory guidance [1]. 

The NephroSSI-PRO comprises 26 items covering the key signs, symptoms, and impacts that have been identified as important to patients with NS, regardless of underlying etiology. It is widely accepted that the underlying causes of NS (FSGS, MCD, MN, or IgAN) are indistinguishable without biopsy [36]. The instrument utilizes an 11-point NRS, due to its simplicity and familiarity, and is supported by considerable literature from other therapeutic areas [37–40], as well as ensuring consistency and ease of responses for patients. A recall period of 24 h was selected based on both regulatory guidance and disease-specific considerations around proteinuria remission and symptom fluctuations [13, 14]. For example, fluid overload in a patient with NS may fluctuate day-to-day based on changes in salt and water intake, or the effect of medications such as diuretics which act within a few hours [41]. Moreover, shorter recall periods (≦ 1 week) have been shown to improve the accuracy and validity of self-reported symptom data [42], and regulatory guidance advises that disease symptoms be assessed closer in time to actual experiences of those symptoms [1, 10]. 

Similar disease-related concepts were reported by both adults and adolescents, with fatigue, swelling and pain-related symptoms identified as the most bothersome across both groups, and indicating the instrument's potential suitability and content vcalidity across age groups for patients with NS aged 12 and older. However, some adolescent patients expressed reservations about completing the NephroSSI-PRO instrument daily for a week, and so adolescent patients may need reminders and help or guidance to complete. This could be achieved in the format of electronic notifications or reminders from the parent/guardian (or both) during a clinical trial.

To the best of our knowledge, this is the first study employing a review of key literature, CE interviews with clinicians, and both CE and CD interviews with adult and adolescent patients with NS caused by varying etiologies. Moreover, because clinical presentation of NS is identical across multiple patterns of kidney pathology and requires biopsy to establish diagnosis, the NephroSSI-PRO represents the first-of-its-kind PRO instrument for patients with NS regardless of underlying etiology which can be used by both adults and adolescents. Previous studies in a similar context were either limited to only FSGS patients [33] or included CE interviews only [29], and have yet to generate a PRO instrument specific to NS. As regulatory guidance recommends clinical outcomes assessment (COA) measures to be age-tailored [10], this study serves as an important first step for content validation of the NephroSSI-PRO in both adult and adolescent age groups before implementation in clinical studies [1]. The next steps for the validation of the NephroSSI-PRO instrument involve its inclusion in Sanofi’s Phase 2a clinical RESULT study (NCT06500702). This next phase will enable item-level analyses to inform potential item reduction, instrument refinement, the definition of a preliminary empirically derived scoring algorithm, determination of response option suitability, and a more accurate estimated completion time for the NephroSSI-PRO. Moreover, psychometric evaluation will be conducted, including assessment of reliability, validity, and responsiveness, as well as estimating meaningful change thresholds (at within-patient, within-group, and between-group levels). In addition, in-trial exit interviews are planned with up to 45 adult and adolescent patients with NS participating in the aforementioned clinical study to further evaluate the content validity of the NephroSSI-PRO [11]. 

### Limitations

This study has several limitations. The literature review was limited as sparse patient-centric empirical research has been published in this target population. Despite this, the interviews with clinicians and patients are of greater importance and provided robust support for the development of the conceptual models. The NephroSSI-PRO has been developed to focus on signs, symptoms, and proximal impacts and did not include more distal impacts such as emotional status or impact on social life; this narrower focus helps minimize patient burden and adopts a focus on clinically relevant endpoints for clinical trials, but investigators more interested in distal impacts may wish to develop further items to measure such concepts. Moreover, conceptual saturation was achieved in adults, but not for adolescents. However, this limitation was anticipated due to the limited CE time due to the hybrid CE/CD interview structure in order to primarily focus on identifying the most bothersome and important symptoms and assessing item-level content validity of the NephroSSI-PRO. While literature suggests that saturation can often be achieved with as few as 10–12 interviews in a relatively homogenous population, it is also acknowledged that conceptual saturation may not always be a reasonable expectation in rare disease populations unless patient experiences are highly uniform. Instead, the goal shifts toward identifying the most important and commonly experienced symptoms and impacts from the patient’s perspective [17]. Although the clinician and adult patient interviews were 10 years before the adolescent interviews, our findings remain pertinent as treatments and overall medical management have not materially changed for these patients over time. Patients interviewed may have been at various stages of disease severity, with different levels of proteinuria, resulting in a heterogeneous perception of symptom severity and impact. Stratified analyses of patients at different stages of the disease were not possible because of the limited sample size; however, such analyses are planned using data from an ongoing Phase 2a clinical trial (NCT06500702) with a much larger sample. While this study included a modest sample that may limit generalizability, the sample size was considered appropriate given this type of research and the rare nature of the disease, and saturation of the concepts was reached for adult patients, suggesting adequate coverage of the most important signs, symptoms and impacts for patients who have lived the longest with NS. Nevertheless, further research involving additional primary glomerulopathies with NS and companion inputs from a larger group of clinicians from different settings may provide deeper insights.

## Conclusion

The NephroSSI-PRO is a novel, disease-specific tool that is the first PRO instrument designed to measure the key signs, symptoms and impacts of NS. It is expected to be a valuable measure in capturing the disease burden in patients with NS and demonstrating treatment value in clinical trials and is included as part of an exploratory endpoint in an ongoing Phase 2a clinical trial (NCT06500702) of approximately 84 patients, whereupon it will undergo formal psychometric validation, including empirically defining a scoring algorithm and subgroup analyses. In-trial exit interviews are also planned to further support its content validity and applicability in the setting of a clinical trial.

## Electronic supplementary material

Below is the link to the electronic supplementary material.

Supplementary Material 1

## Data Availability

Additional data such as study protocol may be shared by the corresponding author upon request.

## Abbreviations

PRO: Patient reported outcomes
NS: Nephrotic syndrome
NephroSSI-PRO: Nephrotic syndrome symptom and impact patient reported outcome
CE: Concept elicitation
FSGS: Focal segmental glomerulosclerosis
MCD: Minimal change disease
MN: Membranous nephropathy
IgAN: Immunoglobulin a nephropathy
HRQoL: Health-related quality of life
US FDA: The United States food and drug administration
CD: Cognitive debriefing
NRS: Numeric rating scale
HRA: Health research associates, Inc
CRO: Clinical research organisation
