# Supplementary material for: Development of the Nephrotic Syndrome Symptom and Impact Patient Reported Outcome (NephroSSI-PRO) measure for use in adults and adolescents with primary glomerulopathies
Source: J Patient Rep Outcomes. 2025 Aug 21;9:106. doi: 10.1186/s41687-025-00937-7 (PMC12370567; doi:10.1186/s41687-025-00937-7)
Supplement: Supplementary file 1 — Supplementary Material 1 [file 41687_2025_937_MOESM1_ESM.docx]

**Supplementary tables**

**Supplementary table 1. Signs, symptoms and impacts identified in literature search**

| **Signs/Symptoms** | **References** |
| --- | --- |
| Edema/Swelling (commonly: eyes,  hands, and legs/feet) | Lin, 2006[19]; National Kidney Foundation, 2012[20]; UNC Kidney Center[21]; The NephCureNephCure Foundation[22] |
| Fatigue | National Kidney Foundation, 2012[20]; Abdel-Kader 2009[23] |
| Weight Gain | The Mayo Clinic, 2012[24] |
| Bodily Pain | Abdel-Kader 2009[23] |
| Poor Appetite | National Kidney Foundation, 2012[20] |
| Dry mouth | Abdel-Kader 2009[23] |
| Dry skin | Abdel-Kader 2009[23] |
| Shortness of breath | National Kidney Foundation, 2012[20]; Abdel-Kader 2009[23] |
|  |  |
| ***Immediate Impacts*** |  |
| Anxiety; Worrying; Feeling  Nervous | Abdel-Kader 2009[23] |
| Sleep Disturbance | Abdel-Kader 2009[23] |
| Sexual Function | Abdel-Kader 2009[23] |
| Irritability | Abdel-Kader 2009[23] |
| Aggressive Behavior | Mehta 1995[25]; Soliday 1999[26] |
|  |  |
| ***General Impacts*** |  |
| Physical function: (climbing stairs, walking short distances, wearing shoes, dressing) | Gipson, 2011[27] |
| Social function | Gipson, 2011[27] |
| Cognitive function | Gipson, 2011[27] |
| Emotional function | Guha 2009[28] |

Note: A review of existing empirical literature was conducted to identify concepts (i.e. signs, symptoms, and impacts) that were important and relevant to patients with FSGS, including prior PRO measures evaluated in patients with FSGS. Articles were selected if they included information on: 1. concepts (signs, symptoms, and impacts) of importance to patients with FSGS from patient’s perspectives; 2. concepts that are important to patients with FSGS from a clinician’s perspective; 3. PRO measures administered to patients with FSGS.

**Supplementary table 2. Signs, symptoms and impacts identified from clinician concept elicitation interviews**

| **Concepts** | **Number of clinicians mentioning concept (N = 4)** |
| --- | --- |
| ***Symptoms*** | |
| **Edema/swelling** | **4** |
| **Weight gain** | **4** |
| **Fatigue/general weakness** | **4** |
| Dyspnea / shortness of breath | 2 |
| Body pain | 1 |
| ***Impacts*** | |
| **Physical functioning** | 4 |
| **Sleep disturbance** | 4 |
| **Emotional health (worry/nervousness/depression)** | 4 |
| Cognitive function | 1 |

(**Bold** = symptoms identified by clinicians as most important to patients.)

Note: Four separate 60-minute interviews were conducted with clinicians (academic physicians) between October – December 2012 who had clinical and research expertise in nephrology with a focus in glomerular diseases. The clinicians described symptoms as generalizable to all patients with NS with primary glomerulopathy.

**Supplementary table 3. Concept elicitation saturation table for the adult population, by participant wave**

| **Concept description** | **Wave 1**  **(n = 4)** | **Wave 2**  **(n = 4)** | **Wave 3**  **(n = 4)** | **Wave 4**  **(n = 4)** |
| --- | --- | --- | --- | --- |
| **Symptoms** | | | |  |
| ***Dryness-related symptoms*** | | | |  |
| Dry mouth |  | X |  |  |
| Itching |  | X |  |  |
| Thirst |  | X |  |  |
| ***Respiratory symptoms*** | | | |  |
| Difficulty in breathing | X |  |  |  |
| Shortness of breath | X |  |  |  |
| Wheezing |  | X |  |  |
| ***Urinary symptoms*** | | | |  |
| Dark/bloody urine | X |  |  |  |
| Foamy urine | X |  |  |  |
| Frequent urination |  | X |  |  |
| ***Digestive symptoms*** | | | |  |
| Appetite | X |  |  |  |
| Nausea and vomiting | X |  |  |  |
| ***Pain symptoms*** | | | |  |
| Cramping | X |  |  |  |
| General body pain | X |  |  |  |
| Headache | X |  |  |  |
| Localized pain in back | X |  |  |  |
| Localized pain in chest | X |  |  |  |
| Localized pain in feet/legs |  | X |  |  |
| ***Edema symptoms*** | | | |  |
| Bloating | X |  |  |  |
| Stretch marks |  |  | X |  |
| Swelling around face/eyes | X |  |  |  |
| Swelling in body/torso | X |  |  |  |
| Swelling in feet/legs | X |  |  |  |
| Swelling in hands/arms |  |  | X |  |
| Tightness |  | X |  |  |
| Water weight |  |  | X |  |
| ***Energy difficulties*** | | | |  |
| General tiredness | X |  |  |  |
| Low stamina |  | X |  |  |
| Low/no energy |  | X |  |  |
| Physical tiredness | X |  |  |  |
| Weakness | X |  |  |  |
| ***Cognitive symptoms*** | | | |  |
| Difficulty concentrating |  |  | X |  |
| Dizziness/lightheadedness | X |  |  |  |
| ***Other symptoms*** | | | |  |
| Blood pressure difficulties |  | X |  |  |
| Numbness/tingling |  |  | X |  |
| ***Number of symptoms emerging by wave, n (%)*** | ***19*** | ***10*** | ***5*** | ***0*** |
| **Impacts** | | | |  |
| ***Physical function limitations and restrictions*** | | | |  |
| General physical function difficulty | X |  |  |  |
| Slowed physical functioning | X |  |  |  |
| Difficulty with stairs | X |  |  |  |
| Difficulty with walking |  | X |  |  |
| ***Difficulty in doing daily activities*** | | | |  |
| General | X |  |  |  |
| Household responsibilities | X |  |  |  |
| Personal care | X |  |  |  |
| Work responsibilities | X |  |  |  |
| ***Social/lifestyle limitations and restrictions*** | | | |  |
| Clothing choice restricted | X |  |  |  |
| Leisure activities restricted |  | X |  |  |
| Relationships affected |  | X |  |  |
| Sexual activities affected |  | X |  |  |
| Social engagements affected | X |  |  |  |
| ***Emotional health*** | | | |  |
| Anger | X |  |  |  |
| Frustration | X |  |  |  |
| Irritability | X |  |  |  |
| Mood swings | X |  |  |  |
| Poor body/self-image | X |  |  |  |
| Sadness/depression | X |  |  |  |
| Worry/fear in general | X |  |  |  |
| Worry/fear of dialysis | X |  |  |  |
| Worry/fear of financial difficulties |  |  | X |  |
| ***Sleep difficulties*** | | | |  |
| Difficulty falling asleep | X |  |  |  |
| Difficulty staying asleep (not due to need to urinate) |  | X |  |  |
| Waking to urinate | X |  |  |  |
| Sleep quality | X |  |  |  |
| Sleep position | X |  |  |  |
| ***Coping behaviors*** | | | |  |
| Dietary restrictions | X |  |  |  |
| General health vigilance |  | X |  |  |
| Germ-vigilant behavior |  |  | X |  |
| Medication | X |  |  |  |
| Outlook | X |  |  |  |
| Sleep |  |  | X |  |
| Social support | X |  |  |  |
| Water consumption | X |  |  |  |
| ***Number of new impacts emerging by wave, n (%)*** | ***26*** | ***6*** | ***3*** | ***0*** |

**Supplementary table 4. Concept Elicitation Saturation Table for the Adolescent population, by participant wave.**

| **Concept description** | **Wave 1**  **(n = 5)** | **Wave 2**  **(n = 5)** | **Wave 3**  **(n = 5)** |
| --- | --- | --- | --- |
| **Symptoms** | | | |
| ***Constitutional Symptoms*** | | | |
| Appetite changes | X |  |  |
| Increased thirst | X |  |  |
| Infection | X |  |  |
| Physical weakness | X |  |  |
| Tiredness/Fatigue | X |  |  |
| Dizziness |  | X |  |
| Excessive sweating |  | X |  |
| Overheated easily |  | X |  |
| Soreness/Tenderness |  | X |  |
| ***Hematological symptoms*** | | | |
| Easily bruised |  | X |  |
| Low iron levels |  | X |  |
| Anemia |  |  | X |
| ***Metabolic and gastrointestinal symptoms*** | | | |
| (Abnormal) weight gain | X |  |  |
| Bloating (abdomen) | X |  |  |
| (Abnormal) weight loss |  | X |  |
| Nausea |  | X |  |
| Constipation |  |  | X |
| Diarrhea |  |  | X |
| ***Pain, general - by location*** | | | |
| Back pain | X |  |  |
| Eye pain | X |  |  |
| Pain in legs | X |  |  |
| Headache | X |  |  |
| Stomachache (pain in abdomen) | X |  |  |
| Pain-related swelling |  | X |  |
| Pain - unspecified location |  | X |  |
| Chest pain |  |  | X |
| Muscle pain |  |  | X |
| Pain in pelvis area |  |  | X |
| ***Swelling - by location*** | | | |
| Swelling in legs, ankles | X |  |  |
| Swelling - whole body | X |  |  |
| Swelling in face and eyes | X |  |  |
| Swelling in abdomen |  | X |  |
| Swelling in arms |  | X |  |
| ***Skin-related symptoms*** | | | |
| Stretchmarks |  | X |  |
| Skin tightness |  |  | X |
| ***Urinary symptoms*** | | | |
| Foamy/frothy urine | X |  |  |
| Pressure when urinating | X |  |  |
| Issues with urinating - unspecified |  | X |  |
| Spilling protein (proteinuria) |  | X |  |
| ***Pulmonary symptoms*** | | | |
| Shortness of breath |  |  | X |
| Fluid in lungs |  |  | X |
| ***Other*** | | | |
| Eating issues - uncertain type |  | X |  |
| *Number of new symptoms emerging by wave, n (%)* | ***17*** | ***16*** | ***9*** |
| **Impacts** | | | |
| ***Impact on activities of daily living (ADL)*** | | | |
| Difficulty dressing |  | X |  |
| Difficulty washing oneself |  | X |  |
| Difficulty feeding oneself |  | X |  |
| General - not specified |  |  | X |
| ***Impact on general activities*** | | | |
| Doing things slower |  | X |  |
| Pushing self to do activities |  | X |  |
| Inability to play |  | X |  |
| Impact on choices - cannot use transportation |  |  | X |
| Impact on choices due to accessibility for needs |  |  | X |
| Impact on hobbies |  |  | X |
| ***Impact on cognitive functioning*** | | | |
| Impact on memory | X |  |  |
| Impact on focus/concentration | X |  |  |
| ***Emotional impact*** | | | |
| Anxiety | X |  |  |
| Apathy | X |  |  |
| Embarrassment | X |  |  |
| Worry about disease | X |  |  |
| Annoyance |  | X |  |
| Depression |  | X |  |
| Feeling happy |  | X |  |
| Mood swings |  | X |  |
| Fear of death |  |  | X |
| Frustration |  |  | X |
| ***Role impact*** | | | |
| Impact on schooling | X |  |  |
| Absenteeism | X |  |  |
| ***Social impact*** | | | |
| Avoidance of social situations | X |  |  |
| Reducing attendance at social events | X |  |  |
| Impact on friendships | X |  |  |
| Making others worry |  | X |  |
| Impact on social functioning |  | X |  |
| Social isolation |  | X |  |
| ***Physical impact*** | | | |
| Impact on vision | X |  |  |
| Impact on driving | X |  |  |
| Inability to practice sports | X |  |  |
| Reduced ability to practice sports | X |  |  |
| Need to rest more |  |  | X |
| Difficulty walking long distance |  |  | X |
| Difficulty walking short distance |  |  | X |
| ***Impact due to compromised immunity*** | | | |
| Relapse because of sickness |  | X |  |
| Need to wear a mask |  | X |  |
| Hygiene restrictions |  | X |  |
| ***Treatment burden*** | | | |
| Keeping track of taking medication |  | X |  |
| ***Impact on diet*** | | | |
| Dietary changes/restrictions | X |  |  |
| ***Psychological impact*** | | | |
| Altered body image | X |  |  |
| Low self-esteem | X |  |  |
| Impact on sexual life | X |  |  |
| ***Other*** | | | |
| Clothing restrictions | X |  |  |
| ***Sleep interference*** | | | |
| Falling asleep early | X |  |  |
| Falling asleep unexpectedly |  | X |  |
| Unable to fall asleep |  | X |  |
| Bad sleep quality |  | X |  |
| ***Number of new impacts emerging by wave, n (%)*** | ***21*** | ***20*** | ***9*** |
